# Multiplex serological screening of wild boar as sentinels of emerging zoonoses: HEV, WNV, and TBEV distribution in Saxony, Germany

**DOI:** 10.1016/j.onehlt.2025.101283

**Published:** 2025-11-19

**Authors:** Lydia Kasper, Balal Sadeghi, Paul Deutschmann, Franziska Stoek, Ute Ziegler, Anne Balkema-Buschmann, Martin H. Groschup, Martin Eiden

**Affiliations:** a Institute of Novel and Emerging Infectious Diseases, Friedrich-Loeffler-Institut, Federal Research Institute for Animal Health, Greifswald, Insel Riems, Germany; b Department of Veterinary Medicine, Saxon State Laboratory of Health and Veterinary Affairs, Jägerstraße 8/10, 01099 Dresden, Germany; c Institute of Diagnostic Virology, Friedrich-Loeffler-Institut, Federal Research Institute for Animal Health, Greifswald, Insel Riems, Germany; d Helmholtz Institute for One Health, Helmholtz-Centre for Infection Research (HZI), Greifswald, Germany

**Keywords:** Hepatitis E virus, Luminex bead-based serology, Multiplex serology, Tick-borne encephalitis virus, West Nile virus, Wild boar

## Abstract

Targeted wildlife surveillance enables early detection of emerging infections and facilitates prevention of wildlife-human spillover events. The Eurasian wild boar (*Sus scrofa*) serves as a valuable indicator species for wildlife surveillance due to its broad European distribution and its ability to adapt to diverse habitats. Wild boar and domestic pigs are the main reservoir for hepatitis E virus (HEV) genotype 3, which can be transmitted to humans mainly through consumption of undercooked meat. Wild boar can also be infected by vector-borne pathogens like the mosquito-transmitted orthoflavivirus West Nile virus (WNV), and the related tick-borne encephalitis virus (TBEV).

To establish an efficient multi-pathogen serological screening system for wild boar surveillance, a multiplex bead-based serological assay was developed for the detection of antibodies against HEV, WNV, and TBEV. Screening of 960 wild boar sera collected in 2023/24 from eight districts in Saxony, Central/Eastern Germany, revealed moderate seropositivity for HEV (24.3 %) and TBEV (33.8 %), and low positivity for WNV (8.4 %). Molecular analysis identified HEV RNA in five individuals, and sequencing followed by phylogenetic analysis showed close relationship to subtype HEV-3i.

These findings demonstrate that wild boar in Saxony are exposed to HEV, WNV, and TBEV, highlighting the value of multiplex serological screening as an effective tool for wildlife-based surveillance of these viruses.

## Highlights

• New multiplex Luminex bead-based assay enables HEV, WNV and TBEV antibody detection.
• Wild boar screening in Saxony revealed low WNV and moderate HEV and TBEV exposure.
• HEV RNA was found in five wild boar, demonstrating infection with HEV genotype 3.
• This study demonstrates wild boar as suitable hosts for zoonotic virus surveillance.

## 1 Introduction

Emerging zoonotic viral diseases pose a significant threat to human and animal health. At the animal-human interface, wildlife populations serve as both reservoirs and sentinels for zoonotic pathogens [1]. Surveillance by wildlife monitoring is thus vital for detecting zoonotic threats, defining risk areas and preventing spillover to domestic animals and humans [2].

Wild boar (*Sus scrofa*) have been identified as key target indicator species for wildlife monitoring of zoonotic diseases [3,4]. These animals are among the most widespread ungulates worldwide and are characterized by a high reproduction rate, a high level of adaptability, and opportunistic feeding habits [5]. Their high abundance and presence in diverse habitats (forests, farmlands, and peri-urban areas) makes exposure to pathogens and their vectors likely and increases potential contacts to domestic animals or humans [6,7]. Wild boar are reservoirs for important zoonotic viruses, bacteria or parasites, including hepatitis E virus (HEV), *Salmonella* spp., *Leptospira* spp., *Trichinella* spp. or *Toxoplasma gondii* [8,9]. Moreover, wild boar can serve as sentinel species for local monitoring of exposure to pathogens such as vector-borne transmitted tick-borne encephalitis virus (TBEV), West Nile virus (WNV), Usutu virus (USUV), *Borrelia burgdorferi* sensu lato or *Rickettsia* spp. [[10], [11], [12], [13]].

Efficient wildlife surveillance can benefit from testing strategies adapted to high-throughput analysis of multiple pathogens in the same assay. To date however, most studies on wild boar rely on separate assays for individual pathogens [8,9]. The main aim of this study was therefore to develop a multiplex serological assay based on the Luminex xMAP technology [14] for parallel analysis of wild boar blood samples for different zoonotic agents. As a proof-of-concept, three zoonotic viruses were selected that are currently under monitoring in Germany. These viruses represent both, pathogens present in wild boar as reservoir hosts with potential fecal-oral transmission (HEV), and vector-borne pathogens with wild boar serving as sentinel hosts (WNV and TBEV).

HEV (*Paslahepevirus balayani*) is one leading cause of acute viral hepatitis worldwide, with 3.3 million symptomatic cases and ∼ 44,000 deaths annually [15]. In Germany, a nation-wide average yearly incidence of 3.91 (2015–2024) is reached, and a high rate of asymptomatic and subclinical cases is anticipated [16,17]. Transmission mainly occurs via the fecal-oral route through contaminated water or food, or via contact with infected animals or their raw meat [17]. HEV genotype 3, the viral subtype predominating in Europe, is mainly carried by domestic pigs (*Sus scrofa domestica*) and wild boar and can infect humans through zoonotic foodborne transmission [[17], [18], [19]].

WNV (*Orthoflavivirus nilense*) circulates between mosquitoes and birds, which act as amplifying hosts [20]. Humans and horses are dead end hosts that can develop illness ranging from mild flu-like up to severe neurological symptoms [20,21]. Through bridge vectors, the virus can spread to a wide range of mammalian species which do not normally contract the disease, including wild boar [22]. WNV is endemic in multiple regions worldwide, including Africa, the Middle East, Australia, Asia, and Europe [20]. Since 2018, the virus has been circulating in Eastern Germany, with regional and temporal variation, and documented infections of birds, humans and horses [23,24].

TBEV (*Orthoflavivirus encephalitidis*) is one of the most common arboviruses in Europe and Asia [25]. In Germany, the virus has been expanding north-eastward, with main risk areas for human infection in German federal states of Bavaria, Baden-Württemberg, Thuringia, Hesse and Saxony [26]. TBEV can cause central nervous system infections in humans, potentially leading to neurological damage or death [27]. The virus circulates between ticks and reservoir hosts such as rodents, insectivores or roe deer, but other vertebrates including wild boar are also susceptible [28].

This study describes the development and validation of a multiplex serological assay for the detection of antibodies against HEV, WNV, and TBEV. The assay was then used to determine the seroprevalences of these three zoonotic viruses in wild boar populations in Saxony, a federal state in Central/Eastern Germany.

## 2 Methods

### 2.1 Samples

The following previously tested reference sera were used for bead-based multiplex binding assay (BMBA) establishment: For the anti-HEV antibody-detecting BMBA, 182 German domestic pig sera (80 positive / 102 negative; according to ID Screen® Hepatitis E Indirect Multi-species ELISA; Innovative Diagnostics, Montpellier, France) were used ([29]; and Pig 18, 19, 23 sera from naturally infected domestic pigs at the Friedrich-Loeffler-Institut). For the BMBAs detecting WNV- and TBEV-specific antibodies, 155 wild boar sera collected 2020/21 in Saxony / Saxony Anhalt, Germany [30] (WNV: 65 positive / 90 negative; TBEV: 38 positive / 117 negative according to virus neutralization tests [30]) were used.

The BMBA screening was done on 960 wild boar sera, collected 2023/24 in Saxony, Germany. Sere were available as residual samples from routine disease monitoring (classical swine fever virus, suid herpesvirus 1 or *Brucella* spp.; [31]) of shot wild boar, provided by the Saxon State Laboratory of Health and Veterinary Affairs. Sampling of shot wild boar was carried out in accordance with the applicable legal regulations (§28a Bundesjagdgesetz (Federal Hunting Act, BJagdG) and §40e Bundesnaturschutzgesetz (Federal Nature Conservation Act, BNatSchG)). Blood was collected post-mortem from the thoracic cavity. Sera were processed by the State Laboratory immediately after reception following standard protocols, and aliquots were stored at −20 °C. Primary quality assessment was performed at the State Laboratory, and only sera suitable for routine diagnostics were shipped frozen to the FLI. No wild boar were purposely sampled for this study.

### 2.2 Molecular analysis

Pooled wild boar sera (960 sera from Saxony, Germany 2023/24; 5 sera per pool) underwent viral RNA extraction via the King Fisher 96 Flex system (Thermo Fisher Scientific, Braunschweig, Germany) with the Nucleo®Mag VET kit (Macherey-Nagel, Düren, Germany), and MS2 bacteriophage RNA as extraction control. HEV and MS2 RNA was detected with HEV ORF3- and MS2-specific RT-qPCR [32], and orthoflavivirus RNA was detected with a pan-flavivirus RT-qPCR [33]. For the HEV-positive pools, RNA from individual sera was extracted using the QIAamp Viral RNA Mini kit (Qiagen, Hilden, Germany) and analyzed with HEV ORF1-specific RT-qPCR [32]. The positive PCR products were Sanger-sequenced (Eurofins, Ebersberg, Germany). Five partial HEV sequences were detected and uploaded to GenBank (https://www.ncbi.nlm.nih.gov/genbank/; accession nos. PQ799465, PQ799466, PQ799467, PQ799468, PQ799469).

For phylogenetic analysis, the five partial HEV sequences (positions 93–345 in HEV isolate GenBank: FJ998008) were aligned with reference HEV sequences in Geneious Prime 2021.0.1. A consensus phylogenetic tree was generated using Neighbor-Joining analysis (Geneious Tree Builder), with avian HEV (NC023425) as the outgroup, genetic distance calculation using the Tamura-Nei method, and resampling with the Bootstrap method (1000 replicates). FigTree version 1.4.4. was used for tree visualization.

### 2.3 Antigen coupling to magnetic microspheres

Recombinant HEV p239 (6 μg), WNV (2.5 μg), or TBEV (5 μg) antigens were coupled to 1.25 × 10^6^ MagPlex® microspheres (Diasorin, Saluggia, Italy) using the Bio-Plex Amine Coupling Kit (Bio-Rad, Feldkirchen, Germany) according to manufacturer's instructions. Protein amounts were optimized for each antigen using positive and negative reference samples. The bead concentration after coupling was determined with an EVE™ cell counter (NanoEntek, Martinsried, Germany).

HEV p239 partial capsid protein (strain KP294371, amino acids 368–606, N-terminal 10× His-tag) was produced in *Escherichia coli* [29,34]*.* WNV non-structural protein 1 (NS1) (strain NY99, amino acids 768–1143) and TBEV NS1 (Ncbi NP_043135.1, amino acids 773–1128) were expressed in mammalian HEK293 cells, incorporating a C-terminal 6× His-tag (The Native Antigen Company, Kidlington, UK).

### 2.4 Bead-based multiplex binding assay (BMBA)

The BMBA serology was performed as described previously [35], with minor modifications. Briefly, 50 μL of coupled beads were added to 96-well plates using a 40 beads/μL dilution in bead buffer (50 % LowCross Buffer, Candor Bioscience, Wangen, Germany, and 50 % Block store buffer, 1 % BSA in PBS). The beads were incubated with 50 μL/well sera or controls (diluted 1:100 if not otherwise stated). Positive sera from naturally HEV-infected domestic pig (Pig 23), from WNV-infected wild boar (WS/20/43) and from TBEV-infected wild boar (WS/21/119) were used as controls. In addition, mouse monoclonal anti-Flavivirus NS1 antibody [D/2/D6/B7] (1:100; Abcam, Cambridge, UK) and mouse monoclonal anti-tick-borne encephalitis virus NS1 (M838) antibody (1:500; The Native Antigen Company, Kidlington, UK) were used as WNV and TBEV positive control, respectively. Detection was done with 50 μL/well mouse anti-human IgG antibody clone HP6017, Fc (1:200; Sigma Aldrich, Taufkirchen, Germany) and goat anti-mouse Alexa 532 antibody (1:100; Thermo Fisher Scientific, Braunschweig, Germany). Results (MFI, mean fluorescence intensity) were recorded using a BioPlex-200 instrument (Bio-Rad, Feldkirchen, Germany), and relative MFI in comparison to positive controls was calculated. All test sera were measured at least once. Sera with low bead counts or other measurement errors in the BMBA assay were repeated at least once, and mean values of the experimental replicates were used for final evaluation.

### 2.5 Enzyme-linked immunosorbent assay (ELISA)

A competition ELISA (cELISA) was used for detecting pan-flavivirus antibodies against the viral envelope protein (ID Screen® Flavivirus Competition, Innovative Diagnostics, Montpellier, France). A blocking ELISA (bELISA) (Ingezim West Nile Compac, Ingenasa, Madrid, Spain) was used for detecting antibodies against domain III of the WNV envelope protein [36].

HEV-specific antibodies were detected using ID Screen® Hepatitis E Indirect Multispecies ELISA (Innovative Diagnostics, Montpellier, France), HEV ELISA 4.0v (MP Diagnostics, Eschwege, Germany), VetLine Hepatitis E Virus ELISA (Gold Standard Diagnostics, Dietzenbach, Germany) or a HEV Inhouse ELISA [29]. ELISAs were performed following manufacturer's instructions. All test sera were measured at least once. Sera with discrepant results between ELISA and BMBA (i.e., positive in one assay but negative in the other) were reanalyzed by ELISA, and mean values of the experimental replicates were used for final evaluation.

### 2.6 Virus neutralization test (VNT)

Selected WNV- or TBEV-positive sera were tested using standard protocols [30], performing separate assays with Vero B4 cells and WNV strain Germany (lineage 2, GenBank accession no. MH924836), Vero 76 cells and USUV strain Germany (Europa 3, GenBank accession no. HE599647), or PK-15 cells and TBEV strain Neudoerfl (GenBank accession no. U27495). All cells were obtained from the Collection of Cell Lines in Veterinary Medicine, Friedrich-Loeffler-Institut, Germany.

### 2.7 Statistical analysis

The serology data were analyzed and visualized in Graphpad Prism 9.5.1., using five-parameter logistic model (5-PL) regression for dilution series analysis and Pearson correlation analysis for comparing HEV BMBA and ELISA data. The seroprevalences were evaluated in R 4.3.1 using the stats base package (exact binomial test for 95 % confidence intervals; Pearson's chi-squared test for regional differences) [37]. Maps were created with Mapchart (https://www.mapchart.net/germany.html), and figures were edited in Inkscape v. 1.4.

BMBA cutoffs, sensitivity and specificity were calculated via Receiver operating characteristic (ROC) curve analysis using the MedCalc software (version 19.6, MedCalc Software Ltd., Ostend, Belgium; https://www.medcalc.org; 2020). The optimal cutoff was set as the point on the ROC curve that yielded the highest Youden index [38] value within the model's output.

## 3 Results

### 3.1 Establishment of a bead-based serology assay for detection of antibodies to hepatitis E virus (HEV), West Nile virus (WNV) and tick-borne encephalitis virus (TBEV)

**Fig. 1:**
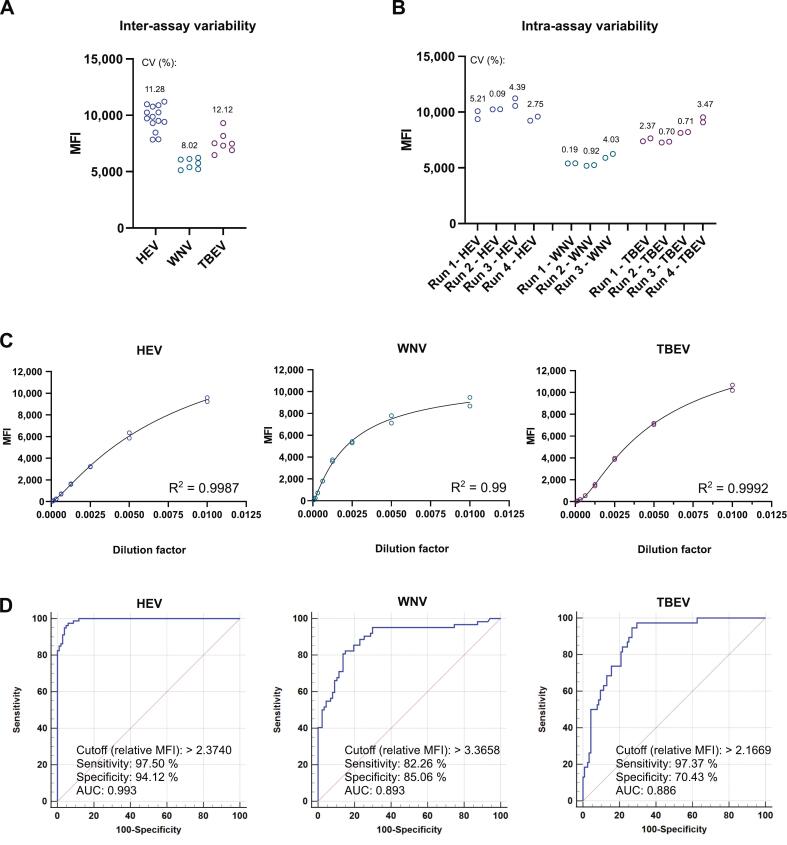
BMBA assay performance characteristics: Inter- and intra-assay variability, dynamic range and receiver operating characteristic (ROC) curve analysis. (A) Mean fluorescence intensity (MFI) of ≥7 independent measurement runs with domestic pig serum Pig 23 (HEV), monoclonal anti-Flavivirus NS1 antibody (WNV), or monoclonal anti-TBEV NS1 antibody (TBEV). CV: coefficient of variation between independent runs. (B) Samples described in A were tested in duplicate in ≥3 independent measurement runs. CVs for duplicates of each run are indicated. (C) Duplicate MFI data from 2-fold serial dilutions (1:100 to 1:12,800) of Pig 23 serum (HEV) or wild boar sera WS/20/43 (WNV) and WS/21/119 (TBEV) with results of a five-parameter logistic model (5-PL) non-linear regression curve and R^2^ values as measure for goodness-of-fit. (D) Shown are ROC curves depicting the area under the curve (AUC; shown by the blue line) as well as the calculated cutoff values, sensitivities, specificities and AUC values. Statistical data on assay performance are summarized in Supplementary Table S3, Supplementary material 1. Analysis of monoplex reactions (one bead-antigen type per assay). (For interpretation of the references to colour in this figure legend, the reader is referred to the web version of this article.)

To enable high-throughput multiplex screening of wild boar sera, existing HEV, WNV, and TBEV Inhouse ELISA protocols [29,30] were transferred to a bead-based multiplex binding assay (BMBA) format. First, assay reproducibility was confirmed by comparing positive control sample measurements across experimental runs (inter-assay variability) and within the same run (intra-assay variability). Inter-assay coefficients of variation (CV) were below 15 %, an accepted maximum inter-assay CV for ELISAs [39] (HEV: 11.3 %, WNV: 8.0 %, TBEV 12.1 %) (Fig. 1A). Intra-assay variability CVs were below 6 % and ranged from 0.1 % to 5.2 % (HEV), 0.2 % to 4 % (WNV) and 0.7 to 3.5 % (TBEV) (Fig. 1B). To assess the assay dynamic range, serial dilutions of positive sera were measured. Data fitted a 5PL non-linear regression model, achieving R^2^ ≥ 0.99. The curves showed a linear progression up to mean fluorescence intensity (MFI) values of ∼4000 (HEV), ∼4000 (WNV) or 8000 (TBEV) (Fig. 1C). A comparison of measurements with single HEV-, WNV- or TBEV-antigen-coupled beads (monoplex) versus combined beads (multiplex) indicated a low cross-reactivity in the multiplex setup (Fig. S1, Supplementary material 2).

Cutoff values, sensitivities and specificities were determined with receiver operating characteristic (ROC) curve analysis using pretested reference sera [29,30]. This analysis established relative MFI cutoffs of 2.374 (HEV), 3.3658 (WNV) and 2.1669 (TBEV) (Fig. 1D). The assay sensitivity / specificity was: 97.5 % / 94.1 % (HEV), 82.3 % / 85.1 % (WNV) and 97.4 % / 70.4 % (TBEV). The corresponding data are compiled in Table S1, Supplementary material 1 (HEV) and Table S2, Supplementary material 1 (WNV and TBEV), while the ROC curve analysis data are summarized in Table S3, Supplementary material 1.

### 3.2 Multiplex bead-based screening for HEV-, WNV- and TBEV-specific antibodies in wild boar

Using the newly established multiplex BMBA, a screening of 960 wild boar sera, collected in eight districts of Saxony (Germany) in 2023/24, was conducted. Raw data are summarized in Table S4, Supplementary material 1. The BMBA screening identified 255 HEV-, 50 WNV-, 302 TBEV- and 33 WNV- & TBEV-positive sera. These results were validated with ELISAs and, for a subset of samples, with VNTs, as described in detail in Supplementary material 2.

For HEV, ELISAs validation confirmed 91.4 % of BMBA HEV-positive sera, and 100 % of selected negative sera (Fig. S2 and S3, Supplementary material 2; Table S4 and S5, Supplementary material 1). Because BMBA-positive sera unconfirmed by ELISA had low relative MFI values (< 12.5), HEV BMBA results from 2.374 (cutoff) to 12.5 should be considered “doubtful” in future screens.

For WNV and TBEV, the BMBA-positive results were strongly supported by two independent flavivirus-specific ELISAs, confirming 96 % of WNV-, 94.7 % of TBEV- and 97 % of WNV- & TBEV-BMBA-positive sera (Fig. S2, Supplementary material 2; Table S4, Supplementary material 1). Among the BMBA-negative sera, a subset tested positive for flavivirus antibodies by ELISA and for Usutu virus (USUV) in VNTs (Supplementary material 2; Table S6, Supplementary material 1). These findings suggest that cross-reactivity or non-WNV or -TBEV flavivirus infections, such as USUV, may account for ELISA-positive but BMBA-negative results. Taken together, the BMBA-based screen, verified by ELISA, reliably detected HEV-, WNV- and TBEV-antibody-positive sera in wild boar sera from Saxony.

### 3.3 Seroprevalence of HEV-, WNV- and TBEV-specific antibodies in wild boar (Saxony)

**Fig. 2:**
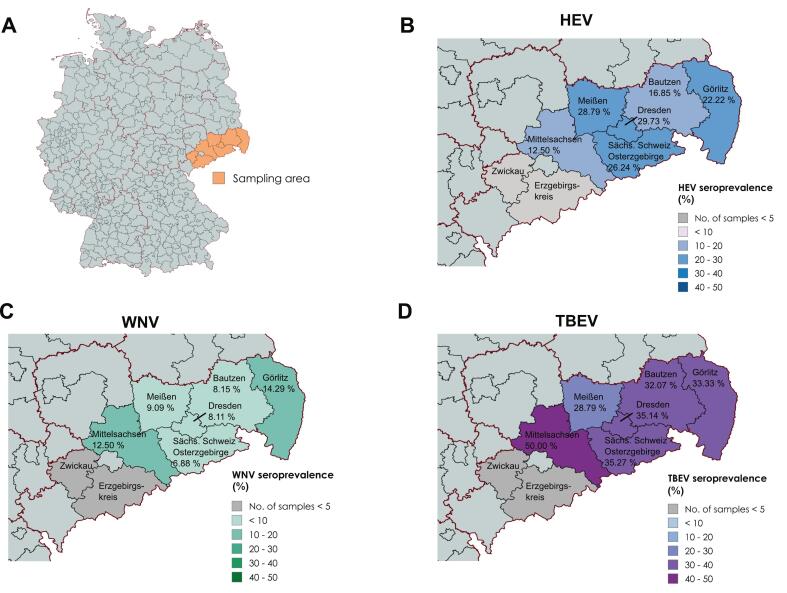
Distribution of seroprevalences of wild boar in different districts of Saxony. (A) Map of Germany with federal state borders (red lines) and district borders (black lines). Wild boar serum samples from eight districts (orange) in the German federal state Saxony were analyzed. HEV- (B), WNV- (C) or TBEV- (D) specific antibodies were detected by BMBA screening and results are depicted as % of positive sera per Saxonian district. Raw data are available in Table S4 and S5, Supplementary material 1. (For interpretation of the references to colour in this figure legend, the reader is referred to the web version of this article.)

The ELISA-confirmed BMBA results were used for assessment of seroprevalences in different districts of Saxony in 2023/24 (Table 1; Fig. 2A). Of 960 sera, 233 were HEV-antibody-positive (24.27 %; CI 95 %: 21.59–27.11). District prevalences ranged from 12.5 % to 29.73 %, but differences between districts were not statistically significant (Pearson's chi-squared test: *P* = 0.09) (Table 1; Fig. 2B).

**Table 1:** Seroprevalence of HEV-, WNV- and TBEV-specific antibodies in wild boar in Saxony (2023/24) based on BMBA screening.

	Number of samples				% Positive (95 % CI)		
	Total	HEV positive	WNV positive	TBEV positive	HEV	WNV	TBEV
All regions	960	233	83	324	24.27 (21.59–27.11)	8.44 (6.76–10.38)	33.75 (30.76–36.84)
Districts:							
Bautzen	184	31	15	59	16.85 (11.74–23.05)	8.15 (4.63–13.09)	32.07 (25.39–39.33)
Dresden city	37	11	3	13	29.73 (15.87–46.98)	8.11 (1.70–21.91)	35.14 (20.21–52.54)
*Erzgebirgskreis*	*3*	*1*	*0*	*2*	*33.33* *(0.84–90.57)*	*0.00* *(0.00–70.76)*	*66.67* *(9.43–99.16)*
Görlitz	126	28	18	42	22.22 (15.3–30.49)	14.29 (8.69–21.63)	33.33 (25.19–42.28)
Meißen	132	38	12	38	28.79 (21.24–37.31)	9.09 (4.79–15.34)	28.79 (21.24–37.31)
Mittelsachsen	8	1	1	4	12.50 (0.32–52.65)	12.50 (0.32–52.65)	50.00 (15.70–84.30)
Sächsische Schweiz-Osterzgebirge	465	122	32	164	26.24 (22.29–30.49)	6.88 (4.75–9.58)	35.27 (30.92–39.8)
*Zwickau*	*1*	*1*	*0*	*0*	*100.00* *(2.50–100)*	*0.00* *(0.00–97.5)*	*0.00* *(0.00–97.5)*
n.s.	4	0	0	2	–	–	–
Pearson's chi-squared test (districts)					Χ-squared = 9.4512, df = 5, p-value = 0.09236	Χ-squared = 7.242, df = 5, *p*-value = 0.2033	Χ-squared = 3.1516, df = 5, p-value = 0.6766

CI: confidence interval; n.s.: not specified; Italics: Districts with <6 tested sera, which were not included into the statistical analyses. Raw data are available in Supplementary Table S4 and S5, Supplementary material 1.

WNV antibodies were found in 81 samples (8.44 %; CI 95 %: 6.76–10.38), with district prevalences ranging from 6.88 % to 14.29 %, also without statistically significant variation between districts (Pearson's chi-squared test: *P* = 0.20) (Table 1; Fig. 2C). Analysis for TBEV detected 324 antibody-positive sera (33.75 %; CI 95 %: 30.76–36.84), with most districts showing 30–35 % prevalence and no statically significant difference across districts (Pearson's chi-squared test: *P* = 0.67) (Table 1; Fig. 2D).

### 3.4 Detection of HEV RNA in five wild boar sera

**Fig. 3:**
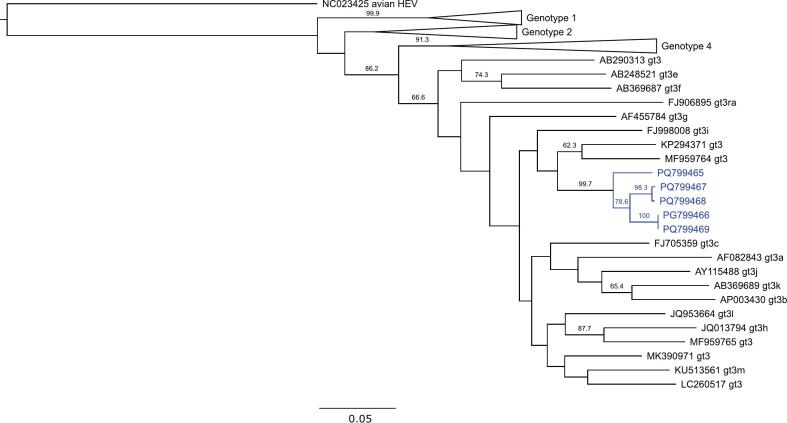
Molecular detection of partial HEV genomes in five wild boar sera. Neighbor-joining phylogenetic tree of the HEV ORF1 gene (*Paslahepevirus balayani* methyltransferase-encoding region; position 93–345 in HEV isolate GenBank: FJ998008), comparing HEV reference sequences of genotypes 1–4 [12], avian HEV and the sequences obtained from five wild boar sera in this study (GenBank Accession numbers PQ799465-PQ799469; subcluster marked in blue). Subtrees for genotype 1, 2, and 4 sequences are collapsed. gt3: genotype 3. Numbers above branches indicate bootstrap support values (only values ≥60 shown). Scale bar indicates substitutions per site. (For interpretation of the references to colour in this figure legend, the reader is referred to the web version of this article.)

Molecular analysis of the 960 wild boar sera detected no orthoflavivirus sequences but identified HEV partial genome sequences in five HEV-seropositive wild boar from Meißen (wild boar 146281135, GenBank Accession no.: PQ799469; wild boar 142926232, GenBank Accession no.: PQ799465), Bautzen (wild boar 146256293, GenBank Accession no.: PQ799466), Görlitz (wild boar 145422371, GenBank Accession no.: PQ799467) and Sächsische Schweiz-Osterzgebirge (wild boar 145423846, GenBank Accession no.: PQ799468). All the sequences belonged to HEV genotype 3. They clustered closely with two wild boar genotype 3 reference sequences from Italy and Germany and with a wild boar / human genotype 3 subtype 3i reference sequence (GenBank accession no.: MF959764, KP294371, FJ998008) [19] (Fig. 3).

## 4 Discussion

The multiplex bead-based serological assay established in this study broadens the methodological tool-box for pathogen-specific antibody screening in wild boar. A serology-based system offers the advantage of identifying both, recent and past infections thereby providing insights into infection dynamics at the population level [40]. Although multiplex-based assays for orthoflavivirus or HEV serology have been described recently, most focus on humans or non-human primates [[41], [42], [43], [44]], or porcine pathogens [45], rather than assessing suids as potential reservoirs or sentinels for zoonotic diseases. The antigens chosen for this study's BMBA assay are established immunogenic markers for the serological diagnosis of HEV and orthoflavivirus infections via ELISA [29,30,46] or multiplex bead-based serology [[41], [42], [43],^45^]. In addition, NS1 antigen-based assays have demonstrated reduced cross-reactivity to other flaviviruses, compared to commercial assays based on the E protein or on whole-virus antigens [47].

This study's BMBA sensitivities and specificities were comparable to ELISAs using the same antigens [29,30]. WNV-/TBEV-BMBA performance values were lower than those for HEV, which may reflect different antibody populations detected during positive/negative classification of reference sera with virus neutralization tests (VNT; the gold standard for differentiating WNV and TBEV antibody responses [48]) versus BMBA measurements of these sera: BMBAs may detect non-neutralizing antibodies missed by VNTs, but may miss antibodies directed against antigens other than NS1. Despite this, BMBA results of screened wild boar sera correlated well with commercial ELISA measurements, showing a high confirmation rate of WNV-/TBEV-BMBA-positives with anti-flavivirus antibody-detecting ELISAs. Flavivirus serology is further complicated by co-circulating orthoflaviviruses, like Usutu virus (USUV) or the flavi-like Alongshan virus, which might elicit cross-reacting antibody responses [24,49,50]. Flavivirus ELISA-positives among BMBA-negative sera suggest the presence of such antibodies to other non-WNV or -TBEV orthoflaviviruses in the tested wild boar sera. Antibodies to USUV are very likely to be present, as VNT-analyses identified such antibodies in four BMBA-negative samples.

The HEV seroprevalence in BMBA-screened wild boar was moderate with 24.27 %. Previous German studies reported similar or higher rates: 43% – 100 % in farmed pigs [51], 33 % in wild boar in Saxony Anhalt (2011) [52], and 23 % - over 50 % in wild boar in Eastern/Northern Germany (2013–2017) [53,54]. These German seroprevalence data align with findings from other European countries demonstrating widespread HEV exposure across the continent: a meta-analysis of seroprevalence data (1990–2020) from 68 studies (57 of which were from European countries) reported a pooled HEV-specific antibody prevalence of 28 % [55]. The HEV RNA prevalence in wild boar sera in this study (5 of 960 wild boar sera; 0.5 %) was lower than in a previous study on wild boar in Saxony (7.3 %) [53]. This previous study, however, mainly used liver for viral RNA isolation, which yields higher virus detection rates than serum testing [34,55]. The RNA sequences from the five HEV-RNA-positive sera showed high similarity to each other (differing by only 0 to 13 nucleotides) and belonged to HEV genotype 3, the main genotype found in infected European humans and wild boar [18]. Wild boar as reservoir hosts, seroconvert following acute and chronic HEV infections, often shedding the virus over prolonged periods without clinical symptoms [56,57]. Taken together, the moderate seroprevalence combined with viral RNA detection in this study indicates HEV exposure and sporadic acute or chronic infection, confirming wild boar as important reservoirs of HEV. This poses potential transmission risks to animals and humans via fecal-to-oral or foodborne routes. In fact, close contact to domestic pigs or wild boar has been discussed as a risk factor for human infection, since forestry or pig sector workers and people in regions with high pig density show higher seroprevalences [[58], [59], [60]]. Studies demonstrating the endemic presence of HEV in wild boar, coupled with the close molecular similarity between HEV strains found in wild boar and humans, underline the zoonotic transmission risk of HEV from wild boar to humans [61,62].

Since the emergence of WNV in Germany in 2018, surveillance has focused on birds and horses, reporting seroprevalences up to 23.3 % in birds [23,24,63] and up to 13.8 % in horses [[64], [65], [66]] in endemic regions of Eastern/Central Germany. The wild boar WNV seroprevalences in Saxony (2023/24) detected in this study were with 6.9–14.3 % lower than in birds but comparable to horses. Holicki et al. [30] reported a higher wild boar WNV seroprevalence of 17.64 % in Saxony / Saxony-Anhalt (2020/21), reflecting possible regional or temporal variation. European wild boar WNV seroprevalence varies widely, from 4.1 % in Czech Republic [67], to 17.6 % in Serbia [12] and 27.5 % in Spain [11]. Although such cross-study comparisons are limited by assay and epidemiological differences, these studies together with the present one, highlight wild boar as WNV hosts in Europe.

The TBEV seroprevalence of 33.75 % detected in this study is similar to previously reported wild boar seroprevalences in 2020/21 in parts of Saxony (Nordsachsen, 30.9 %) or Saxony Anhalt (Anhalt-Bitterfeld, 27.96 %), though the overall seroprevalence in that study was lower (12.91 %) [30]. An earlier study detected 10.5 % TBEV seroprevalence among (mainly) wild boar in Saxony (2011−2013), with high seroprevalence in Meißen (23 %) and Dresden (18 %) [10]. Together, these data confirm that wild boar in Saxony have been exposed to TBEV for more than a decade, with higher seroprevalences in recent years, potentially indicating ongoing TBEV expansion in that region. Neighboring European countries have conducted similar seroprevalence studies on TBEV in wild boar. Compared to the present study, TBEV seroprevalences reported in Eastern France (2.9 %) and Northern Belgium (9.27 %) were lower, aligning with the relatively low public health risk of TBEV in these regions [[68], [69], [70]]. No orthoflavivirus RNA was found in wild boar sera, consistent with previous findings of no (WNV) or only 0.1 % (TBEV) RNA-positive sera among wild boar in Saxony / Saxony Anhalt [30]. This indicates that most WNV- or TBEV-infected wild boar exhibit no, mild or short-term viremia. Although infected pigs and wild boar often seroconvert and produce virus-specific antibodies, they are not considered as amplifying hosts for WNV or TBEV [28,71]. However, as wild boar are mainly hunted in winter, after the peak vector-borne transmission season, acute infections from summer may have been missed in the current study setup.

## 5 Conclusions and outlook

This work has established and validated a bead-based multiplex binding assay (BMBA) screening system for detection of antibodies to HEV, WNV and TBEV in wild boar. The current assay enables efficient simultaneous screening for three zoonotic viruses, with the potential of future expansion to include additional pathogens relevant to wild boar [[8], [9], [10], [11], [12], [13]]. The current study adds recent seroprevalence and HEV sequence data to wildlife surveillance in Germany. The presented data aligns with previous studies demonstrating ongoing exposure of wild boar in Saxony to HEV, WNV, and TBEV and confirming their suitability as hosts for surveillance. BMBA-based wild boar monitoring can complement ongoing wildlife surveillance programs such as bird monitoring for WNV, helping to track virus spread and providing an early warning system for new transmission events. Future wild boar monitoring would benefit from expanding BMBA-based serological testing to include samples from other German federal states and different sampling years, to enhance our understanding of pathogen dynamics and enable comprehensive analysis of virus exposure and spread across regions and over time.

## CRediT authorship contribution statement

**Lydia Kasper:** Writing – review & editing, Writing – original draft, Visualization, Validation, Methodology, Investigation, Formal analysis, Data curation, Conceptualization. **Balal Sadeghi:** Writing – review & editing, Visualization, Validation, Formal analysis, Data curation, Conceptualization. **Paul Deutschmann:** Writing – review & editing, Resources. **Franziska Stoek:** Writing – review & editing, Conceptualization. **Ute Ziegler:** Writing – review & editing, Investigation, Formal analysis, Conceptualization. **Anne Balkema-Buschmann:** Writing – review & editing, Resources, Methodology, Conceptualization. **Martin H. Groschup:** Writing – review & editing, Funding acquisition, Conceptualization. **Martin Eiden:** Writing – review & editing, Writing – original draft, Validation, Supervision, Resources, Methodology, Data curation, Conceptualization.

## Funding sources

The study is part of the Seed Grant for One Health Surveillance 2023 project “Innovative Diagnostic Pipelines for One Health Surveillance (InnoDia)” funded by the “Initialisierungs- und Vernetzungsfonds für Infektionsforschung” and managed by the Helmholtz Institute for One Health.

## Declaration of competing interest

The authors declare that they have no known competing financial interests or personal relationships that could have appeared to influence the work reported in this paper.

## Data Availability

All study data are included in the manuscript or supplement. Partial HEV sequences were deposited in GenBank (https://www.ncbi.nlm.nih.gov/genbank/) and will be available upon manuscript acceptance.

## Appendix A Supplementary data

Supplementary material 1 Image 1

Supplementary material 2 Image 2

## References

[1] Halliday J.E.B., Meredith A.L., Knobel D.L., Shaw D.J., Bronsvoort B.M.D.C., Cleaveland S. (2007). A framework for evaluating animals as sentinels for infectious disease surveillance. J. R. Soc. Interface.

[2] Lawson B., Neimanis A., Lavazza A., López-Olvera J.R., Tavernier P., Billinis C., Duff J.P., Mladenov D.T., Rijks J.M., Savić S., Wibbelt G., Ryser-Degiorgis M.-P., Kuiken T. (2021). How to start up a National Wildlife Health Surveillance Programme. Animals (Basel).

[3] Cardoso B., García-Bocanegra I., Acevedo P., Cáceres G., Alves P.C., Gortázar C. (2022). Stepping up from wildlife disease surveillance to integrated wildlife monitoring in Europe. Res. Vet. Sci..

[4] Barroso P., Relimpio D., Zearra J.A., Cerón J.J., Palencia P., Cardoso B., Ferreras E., Escobar M., Cáceres G., López-Olvera J.R., Gortázar C. (2023). Using integrated wildlife monitoring to prevent future pandemics through one health approach. One Health.

[5] Ferrara G., Tejeda C. (2024). Editorial: wildlife-domestic animal interface: threat or sentinel?. Front. Vet. Sci..

[6] Massei G., Kindberg J., Licoppe A., Gačić D., Šprem N., Kamler J., Baubet E., Hohmann U., Monaco A., Ozoliņš J., Cellina S., Podgórski T., Fonseca C., Markov N., Pokorny B., Rosell C., Náhlik A. (2015). Wild boar populations up, numbers of hunters down? A review of trends and implications for Europe. Pest Manag. Sci..

[7] Miettinen E., Melin M., Holmala K., Meller A., Väänänen V.-M., Huitu O., Kunnasranta M. (2023). Home ranges and movement patterns of wild boars (Sus scrofa) at the northern edge of the species’ distribution range. Mamm. Res..

[8] Abrantes A.C., Vieira-Pinto M. (2023). 15 years overview of European zoonotic surveys in wild boar and red deer: a systematic review. One Health.

[9] Ruiz-Fons F. (2017). A review of the current status of relevant zoonotic pathogens in wild swine (Sus scrofa) populations: changes modulating the risk of transmission to humans. Transbound. Emerg. Dis..

[10] Balling A., Plessow U., Beer M., Pfeffer M. (2014). Prevalence of antibodies against tick-borne encephalitis virus in wild game from Saxony, Germany. Ticks Tick Borne Dis..

[11] Casades-Martí L., Cuadrado-Matías R., Peralbo-Moreno A., Baz-Flores S., Fierro Y., Ruiz-Fons F. (2023). Insights into the spatiotemporal dynamics of West Nile virus transmission in emerging scenarios. One Health.

[12] Escribano-Romero E., Lupulović D., Merino-Ramos T., Blázquez A.-B., Lazić G., Lazić S., Saiz J.-C., Petrović T. (2015). West Nile virus serosurveillance in pigs, wild boars, and roe deer in Serbia. Vet. Microbiol..

[13] Grassi L., Drigo M., Zelená H., Pasotto D., Cassini R., Mondin A., Franzo G., Tucciarone C.M., Ossola M., Vidorin E., Menandro M.L. (2023). Wild ungulates as sentinels of flaviviruses and tick-borne zoonotic pathogen circulation: an Italian perspective. BMC Vet. Res..

[14] Bernhard O.K., Mathias R.A., Barnes T.W., Simpson R.J. (2011). A fluorescent microsphere-based method for assay of multiple analytes in plasma. Methods Mol. Biol..

[15] Devarbhavi H., Asrani S.K., Arab J.P., Nartey Y.A., Pose E., Kamath P.S. (2023). Global burden of liver disease: 2023 update. J. Hepatol..

[16] Robert Koch Institute (2025). Survstat@RKI 2.0: HEV Incidence in Germany 2015–2024. https://survstat.rki.de.

[17] Zahmanova G., Takova K., Tonova V., Koynarski T., Lukov L.L., Minkov I., Pishmisheva M., Kotsev S., Tsachev I., Baymakova M., Andonov A.P. (2023). The re-emergence of hepatitis E virus in Europe and vaccine development. Viruses.

[18] Pavio N., Doceul V., Bagdassarian E., Johne R. (2017). Recent knowledge on hepatitis E virus in Suidae reservoirs and transmission routes to human. Vet. Res..

[19] Smith D.B., Izopet J., Nicot F., Simmonds P., Jameel S., Meng X.-J., Norder H., Okamoto H., van der Poel W.H.M., Reuter G., Purdy M.A. (2020). Update: proposed reference sequences for subtypes of hepatitis E virus (species Orthohepevirus A). J. Gen. Virol..

[20] Habarugira G., Suen W.W., Hobson-Peters J., Hall R.A., Bielefeldt-Ohmann H. (2020). West Nile virus: an update on pathobiology, epidemiology, diagnostics, control and “One Health” implications. Pathogens.

[21] Castillo-Olivares J., Wood J. (2004). West Nile virus infection of horses. Vet. Res..

[22] Root J.J., Bosco-Lauth A.M. (2019). West Nile virus associations in wild mammals: an update. Viruses.

[23] Ziegler U., Lühken R., Keller M., Cadar D., van der Grinten E., Michel F., Albrecht K., Eiden M., Rinder M., Lachmann L., Höper D., Vina-Rodriguez A., Gaede W., Pohl A., Schmidt-Chanasit J., Groschup M.H. (2019). West Nile virus epizootic in Germany, 2018. Antivir. Res..

[24] Schopf F., Sadeghi B., Bergmann F., Fischer D., Rahner R., Müller K., Günther A., Globig A., Keller M., Schwehn R., Guddorf V., Reuschel M., Fischer L., Krone O., Rinder M., Schütte K., Schmidt V., Heenemann K., Schwarzer A., Fast C., Sauter-Louis C., Staubach C., Lühken R., Schmidt-Chanasit J., Brandes F., Lierz M., Korbel R., Vahlenkamp T.W., Groschup M.H., Ziegler U. (2024). Circulation of West Nile virus and Usutu virus in birds in Germany, 2021 and 2022. Infect. Dis. (Lond.).

[25] Im J.H., Baek J.-H., Durey A., Kwon H.Y., Chung M.-H., Lee J.-S. (2020). Geographic distribution of tick-borne encephalitis virus complex. J. Vector Borne Dis..

[26] Robert Koch Institute (2025). Epidemiologisches Bulletin 09/2025: TBEV Risk Areas in Germany. https://www.rki.de/DE/Aktuelles/Publikationen/Epidemiologisches-Bulletin/2025/09_25.html.

[27] Pustijanac E., Buršić M., Talapko J., Škrlec I., Meštrović T., Lišnjić D. (2023). Tick-borne encephalitis virus: a comprehensive review of transmission, pathogenesis, epidemiology, clinical manifestations, diagnosis, and prevention. Microorganisms.

[28] Michelitsch A., Wernike K., Klaus C., Dobler G., Beer M. (2019). Exploring the reservoir hosts of tick-borne encephalitis virus. Viruses.

[29] Suluku R., Jabaty J., Fischer K., Diederich S., Groschup M.H., Eiden M. (2024). Hepatitis E Seroprevalence and detection of genotype 3 strains in domestic pigs from Sierra Leone collected in 2016 and 2017. Viruses.

[30] Holicki C.M., Ziegler U., Gaede W., Albrecht K., Hänske J., Walraph J., Sadeghi B., Groschup M.H., Eiden M. (2025). Tracking WNV transmission with a combined dog and wild boar surveillance system. Sci. Rep..

[31] Sächsische Tierseuchenkasse (2023). Jahresbericht. https://www.tsk-sachsen.de/documents/Jahresberichte/2024/Jahresbericht_Endfassung.pdf.

[32] Vina-Rodriguez A., Schlosser J., Becher D., Kaden V., Groschup M.H., Eiden M. (2015). Hepatitis E virus genotype 3 diversity: phylogenetic analysis and presence of subtype 3b in wild boar in Europe. Viruses.

[33] Vina-Rodriguez A., Sachse K., Ziegler U., Chaintoutis S.C., Keller M., Groschup M.H., Eiden M. (2017). A novel Pan-Flavivirus detection and identification assay based on RT-qPCR and microarray. Biomed. Res. Int..

[34] Dähnert L., Aliabadi E., Fast C., Hrabal I., Schröder C., Behrendt P., Protzer U., Groschup M.H., Eiden M. (2024). Immunisation of pigs with recombinant HEV vaccines does not protect from infection with HEV genotype 3. One Health.

[35] Mbu’u C.M., Gontao P., Wade A., Penning M., Sadeghi B., Mbange A.E., LeBreton M., Kamdem S.L.S., Stoek F., Groschup M.H., Mbacham W.F., Balkema-Buschmann A. (2025). Serological and molecular analysis of henipavirus infections in synanthropic fruit bat and rodent populations in the Centre and North regions of Cameroon (2018-2020). BMC Vet. Res..

[36] Sotelo E., Llorente F., Rebollo B., Camuñas A., Venteo A., Gallardo C., Lubisi A., Rodríguez M.J., Sanz A.J., Figuerola J., Jiménez-Clavero M.Á. (2011). Development and evaluation of a new epitope-blocking ELISA for universal detection of antibodies to West Nile virus. J. Virol. Methods.

[37] R Core Team (2025). https://www.R-project.org/.

[38] Youden W.J. (1950). Index for rating diagnostic tests. Cancer.

[39] Food and Drug Administration (2018). Bioanalytical Method Validation Guidance for Industry. https://www.fda.gov/regulatory-information/search-fda-guidance-documents/bioanalytical-method-validation-guidance-industry.

[40] Alter G., Seder R. (2020). The power of antibody-based surveillance. N. Engl. J. Med..

[41] Tyson J., Tsai W.-Y., Tsai J.-J., Mässgård L., Stramer S.L., Lehrer A.T., Nerurkar V.R., Wang W.-K. (2019). A high-throughput and multiplex microsphere immunoassay based on non-structural protein 1 can discriminate three flavivirus infections. PLoS Negl. Trop. Dis..

[42] Raulino R., Thaurignac G., Butel C., Villabona-Arenas C.J., Foe T., Loul S., Ndimbo-Kumugo S.-P., Mbala-Kingebeni P., Makiala-Mandanda S., Ahuka-Mundeke S., Kerkhof K., Delaporte E., Ariën K.K., Foulongne V., Mpoudi Ngole E., Peeters M., Ayouba A. (2021). Multiplex detection of antibodies to chikungunya, O’nyong-nyong, Zika, dengue, West Nile and Usutu viruses in diverse non-human primate species from Cameroon and the Democratic Republic of Congo. PLoS Negl. Trop. Dis..

[43] Merbah M., Wollen-Roberts S., Shubin Z., Li Y., Bai H., Dussupt V., Mendez-Rivera L., Slike B., Krebs S.J., Modjarrad K., Michael N.L., Rolland M. (2020). A high-throughput multiplex assay to characterize flavivirus-specific immunoglobulins. J. Immunol. Methods.

[44] Beck C., Desprès P., Paulous S., Vanhomwegen J., Lowenski S., Nowotny N., Durand B., Garnier A., Blaise-Boisseau S., Guitton E., Yamanaka T., Zientara S., Lecollinet S. (2015). A high-performance multiplex immunoassay for Serodiagnosis of flavivirus-associated neurological diseases in horses. Biomed. Res. Int..

[45] Aira C., Penning M., Eiden M., Balkema-Buschmann A., Blome S., Strutzberg-Minder K., López L., Rueda P., Sastre P. (2022). A multiplex assay for the detection of antibodies to relevant swine pathogens in serum. Transbound. Emerg. Dis..

[46] Mora-Cárdenas E., Aloise C., Faoro V., Knap Gašper N., Korva M., Caracciolo I., D’Agaro P., Avšič-Županc T., Marcello A. (2020). Comparative specificity and sensitivity of NS1-based serological assays for the detection of flavivirus immune response. PLoS Negl. Trop. Dis..

[47] Ceconi M., Ariën K.K., Delputte P. (2024). Diagnosing arthropod-borne flaviviruses: non-structural protein 1 (NS1) as a biomarker. Trends Microbiol..

[48] Yeh J.-Y., Lee J.-H., Park J.-Y., Seo H.-J., Moon J.-S., Cho I.-S., Kim H.-P., Yang Y.-J., Ahn K.-M., Kyung S.-G., Choi I.-S., Lee J.-B. (2012). A diagnostic algorithm to serologically differentiate West Nile virus from Japanese encephalitis virus infections and its validation in field surveillance of poultry and horses. Vector Borne Zoonot. Dis..

[49] Da Gomes Silva P., Seixas Dos Reis J.A., Nogueira Rodrigues M., Da Silva Ardaya Q., Mesquita J.R. (2023). Serological cross-reactivity in zoonotic flaviviral infections of medical importance. Antibodies (Basel).

[50] Ebert C.L., Söder L., Kubinski M., Glanz J., Gregersen E., Dümmer K., Grund D., Wöhler A.-S., Könenkamp L., Liebig K., Knoll S., Hellhammer F., Topp A.-K., Becher P., Springer A., Strube C., Nagel-Kohl U., Nordhoff M., Steffen I., Bauer B.U., Ganter M., Feige K., Becker S.C., Boelke M. (2023). Detection and characterization of Alongshan virus in ticks and tick saliva from lower Saxony, Germany with serological evidence for viral transmission to game and domestic animals. Microorganisms.

[51] Dremsek P., Joel S., Baechlein C., Pavio N., Schielke A., Ziller M., Dürrwald R., Renner C., Groschup M.H., Johne R., Krumbholz A., Ulrich R.G. (2013). Hepatitis E virus seroprevalence of domestic pigs in Germany determined by a novel in-house and two reference ELISAs. J. Virol. Methods.

[52] Denzin N., Borgwardt J. (2013). Vorkommen und geografische Verbreitung von Antikörpern gegen Hepatitis E-virus beim Wildschwein in Sachsen-Anhalt (2011). Berl. Munch. Tierarztl. Wochenschr..

[53] Schotte U., Martin A., Brogden S., Schilling-Loeffler K., Schemmerer M., Anheyer-Behmenburg H.E., Szabo K., Müller-Graf C., Wenzel J.J., Kehrenberg C., Binder A., Klein G., Johne R. (2022). Phylogeny and spatiotemporal dynamics of hepatitis E virus infections in wild boar and deer from six areas of Germany during 2013-2017. Transbound. Emerg. Dis..

[54] Anheyer-Behmenburg H.E., Szabo K., Schotte U., Binder A., Klein G., Johne R. (2017). Hepatitis E virus in wild boars and spillover infection in red and roe deer, Germany, 2013-2015. Emerg. Infect. Dis..

[55] Fanelli A., Tizzani P., Buonavoglia D. (2021). A systematic review and meta-analysis of hepatitis E virus (HEV) in wild boars. Res. Vet. Sci..

[56] Schlosser J., Eiden M., Vina-Rodriguez A., Fast C., Dremsek P., Lange E., Ulrich R.G., Groschup M.H. (2014). Natural and experimental hepatitis E virus genotype 3-infection in European wild boar is transmissible to domestic pigs. Vet. Res..

[57] Schlosser J., Vina-Rodriguez A., Fast C., Groschup M.H., Eiden M. (2015). Chronically infected wild boar can transmit genotype 3 hepatitis E virus to domestic pigs. Vet. Microbiol..

[58] Dremsek P., Wenzel J.J., Johne R., Ziller M., Hofmann J., Groschup M.H., Werdermann S., Mohn U., Dorn S., Motz M., Mertens M., Jilg W., Ulrich R.G. (2012). Seroprevalence study in forestry workers from eastern Germany using novel genotype 3- and rat hepatitis E virus-specific immunoglobulin G ELISAs. Med. Microbiol. Immunol..

[59] Krumbholz A., Joel S., Dremsek P., Neubert A., Johne R., Dürrwald R., Walther M., Müller T.H., Kühnel D., Lange J., Wutzler P., Sauerbrei A., Ulrich R.G., Zell R. (2014). Seroprevalence of hepatitis E virus (HEV) in humans living in high pig density areas of Germany. Med. Microbiol. Immunol..

[60] Janssens H., Delameillieure L., Jonckheere S., van Houtte F., Meuleman P., Geens T. (2025). Higher hepatitis E Seroprevalence in workers in the pig sector in Flanders, Belgium: results from a Seroprevalence case-control study. Zoonoses Public Health.

[61] Wang B., Meng X.-J. (2021). Hepatitis E virus: host tropism and zoonotic infection. Curr. Opin. Microbiol..

[62] Ruiz-Ponsell L., Monastiri A., López-Roig M., Sauleda S., Bes M., Mentaberre G., Escobar-González M., Costafreda M.I., López-Olvera J.R., Serra-Cobo J. (2024). Endemic maintenance of human-related hepatitis E virus strains in synurbic wild boars, Barcelona Metropolitan Area, Spain. Sci. Total Environ..

[63] Ziegler U., Bergmann F., Fischer D., Müller K., Holicki C.M., Sadeghi B., Sieg M., Keller M., Schwehn R., Reuschel M., Fischer L., Krone O., Rinder M., Schütte K., Schmidt V., Eiden M., Fast C., Günther A., Globig A., Conraths F.J., Staubach C., Brandes F., Lierz M., Korbel R., Vahlenkamp T.W., Groschup M.H. (2022). Spread of West Nile virus and Usutu virus in the German Bird Population, 2019-2020. Microorganisms.

[64] Gothe L.M.R., Ganzenberg S., Ziegler U., Obiegala A., Lohmann K.L., Sieg M., Vahlenkamp T.W., Groschup M.H., Hörügel U., Pfeffer M. (2023). Horses as sentinels for the circulation of flaviviruses in eastern-Central Germany. Viruses.

[65] Ganzenberg S., Sieg M., Ziegler U., Pfeffer M., Vahlenkamp T.W., Hörügel U., Groschup M.H., Lohmann K.L. (2022). Seroprevalence and risk factors for equine West Nile virus infections in eastern Germany, 2020. Viruses.

[66] Bergmann F., Trachsel D.S., Stoeckle S.D., Bernis Sierra J., Lübke S., Groschup M.H., Gehlen H., Ziegler U. (2022). Seroepidemiological survey of West Nile virus infections in horses from Berlin/Brandenburg and North Rhine-Westphalia, Germany. Viruses.

[67] Hubálek Z., Juricová Z., Straková P., Blazejová H., Betásová L., Rudolf I. (2017). Serological survey for West Nile virus in wild artiodactyls, southern Moravia (Czech Republic). Vector Borne Zoonot. Dis..

[68] Beerlage-de Jong N., Blanford J. (2025). Mapping the risk of tick-borne encephalitis in Europe for informed vaccination decisions. J. Travel Med..

[69] Adjadj N.R., Vervaeke M., Sohier C., Cargnel M., de Regge N. (2022). Tick-borne encephalitis virus prevalence in sheep, wild boar and ticks in Belgium. Viruses.

[70] Bournez L., Umhang G., Faure E., Boucher J.-M., Boué F., Jourdain E., Sarasa M., Llorente F., Jiménez-Clavero M.A., Moutailler S., Lacour S.A., Lecollinet S., Beck C. (2019). Exposure of wild ungulates to the Usutu and tick-borne encephalitis viruses in France in 2009-2014: evidence of undetected flavivirus circulation a decade ago. Viruses.

[71] Teehee M.L., Bunning M.L., Stevens S., Bowen R.A. (2005). Experimental infection of pigs with West Nile virus. Arch. Virol..

