# Supplementary material for: Multiplex serological screening of wild boar as sentinels of emerging zoonoses: HEV, WNV, and TBEV distribution in Saxony, Germany
Source: One Health. 2025 Nov 19;21:101283. doi: 10.1016/j.onehlt.2025.101283 (PMC12685552; doi:10.1016/j.onehlt.2025.101283)
Supplement: Supplementary file 2 — Supplementary material 2 [file mmc2.docx]

***Multiplex serological screening of wild boar as sentinels of emerging zoonoses: HEV, WNV, and TBEV distribution in Saxony, Germany***

*Lydia Kasper, Balal Sadeghi, Paul Deutschmann, Franziska Stoek, Ute Ziegler, Anne Balkema-Buschmann, Martin H. Groschup and Martin Eiden*

**Supplementary material 2:**

**Bead-based multiplex binding assay (BMBA) screen validation**

**Comparison of monoplex and multiplex assay setups**

BMBA measurements of control sera with single (monoplex) *versus* combined (multiplex) HEV‑, WNV-, or TBEV-antigen-coupled beads detected a low average difference of 5.5% between monoplex and multiplex measurements, indicating low cross-reactivity in the multiplex setup (Fig. S1).


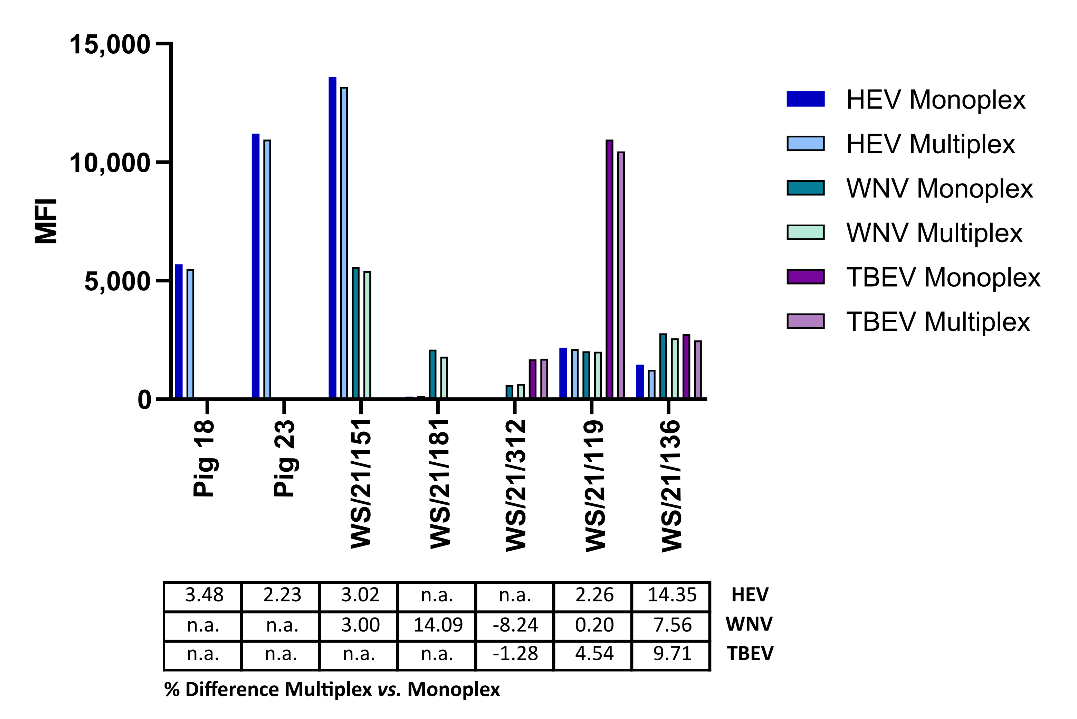


**Figure S1: Comparison of monoplex and multiplex bead-based assays.** Mean fluorescence intensity values (MFI) of domestic pig or wild boar control sera. Sera had been pre-analyzed for HEV-antibodies with ID Screen® Hepatitis E Indirect Multi-species ELISA (domestic pig 18 and 23; both HEV positive; Table S1, Supplementary material 1), or for WNV- or TBEV-neutralizing antibodies with VNT (wild boar WS21/151 and 21/181 WNV-positive; WS21/312, 21/119, 21/136 WNV- and TBEV-positive [1]). The sera were tested in bead-based monoplex assays with single HEV-, WNV- or TBEV-antigen-coupled beads or in a multiplex reaction which combined all three bead types. The table shows the percentage difference between monoplex and multiplex MFI values. n.a. not available (% difference not calculated for samples with MFI values < 200).

**Validation of BMBA data with ELISA and VNT**

For validation of the BMBA screening of 960 wild boar from Saxony (Germany, 2023/24), positive and randomly selected negative samples were first re-tested with commercial ELISAs as outlined in Fig. S2. A subset of WNV/TBEV BMBA-positive or -negative sera was further subjected to virus neutralization test (VNT) analysis for WNV-, USUV- and TBEV-specific neutralizing antibodies (see below).

All tested HEV BMBA-negative sera were also negative in an ID Screen HEV ELISA. Of the 255 BMBA HEV-positive sera, 207 (81.2 %) were confirmed positive in the ID Screen HEV ELISA while 48 were doubtful or negative (Fig. S2; Table S5, Supplementary material 1).


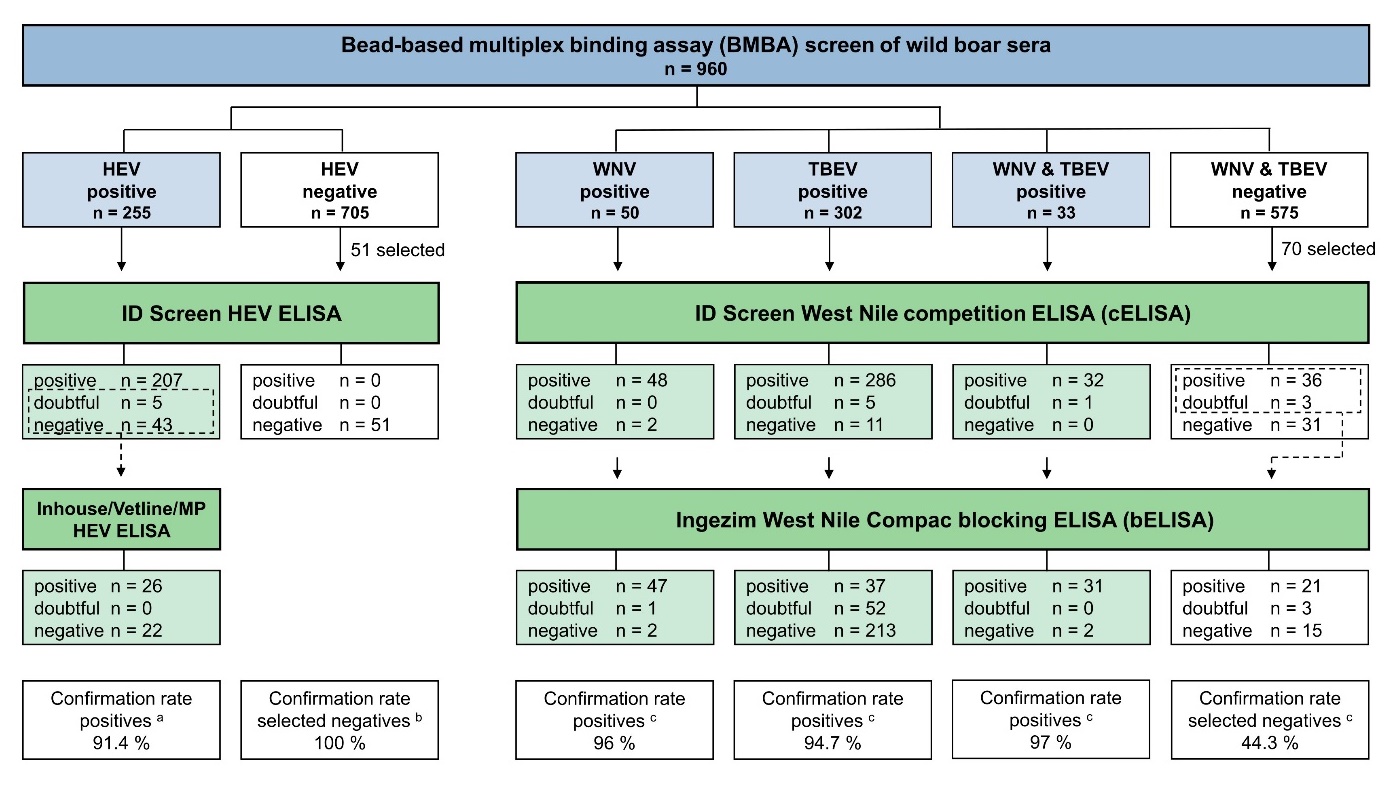


**Figure S2.** **Outline of BMBA screen and ELISA validation of wild boar sera.** 960 wild boar sera were screened for HEV-, WNV- and TBEV-specific antibodies using the bead-based multiplex binding assay (BMBA). All BMBA-positive and selected BMBA-negative sera were further analyzed by ELISA. Confirmation rates of BMBA positives or negatives by ELISA are indicated.

^a^ % of BMBA-positives confirmed by HEV ID Screen, Inhouse, Vetline, and/or MP-ELISA; ^b^ % of selected BMBA-negatives confirmed by HEV ID Screen ELISA; ^c^ BMBA-positives / selected BMBA-negatives confirmed by cELISA. Raw data are available in Table S4 and S5, Supplementary material 1.

HEV BMBA and ID Screen ELISA data showed a positive correlation (Pearson correlation analysis; r = 0.67; *P* < .0001), with strong agreement between assays for sera with high BMBA fluorescence. In contrast, most unconfirmed HEV BMBA-positives showed low fluorescence in the BMBA, suggesting low antibody levels (Fig. S3). Re-testing of these sera with three additional HEV ELISAs (MP Diagnostics 4.0v, VetLine or Inhouse ELISA [2]) detected 26 of the 48 ambiguous samples as positive (Table S5, Supplementary material 1), yielding an overall confirmation rate of 91.4 % of the HEV BMBA-positives by ELISA. The 22 BMBA-positive sera that remained unconfirmed by all four ELISAs had a low relative MFI of < 12.5. Therefore, HEV BMBA results between 2.374 (cutoff; ROC curve analysis) and 12.5 should be classified as "doubtful" in future screens.


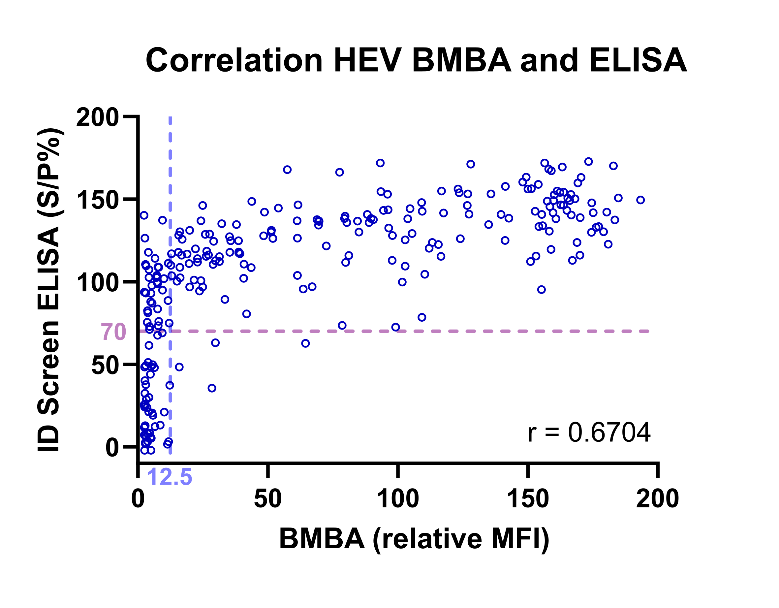


**Figure S3: Correlation of HEV BMBA and ELISA data**. HEV BMBA and ID Screen ELISA data of HEV BMBA-positive sera (MFI > 2.374). Data were subjected to a Pearson correlation analysis, with the output r = 0.6704 (CI 95 %: 0.5967-0.7329); df = 254; *P* < .0001. Raw data are available in Table S4, Supplementary material 1. Relative MFI (mean fluorescence intensity): MFI [sample]/MFI [positive control] * 100; S/P%: OD sample/ OD positive control * 100). Dashed pink line indicates ELISA cutoff (S/P% ≥ 70 = positive). Dashed light blue line indicates the relative MFI value 12.5, below which BMBA values should be considered as “doubtful”.

To validate the WNV and TBEV BMBA results, the sera were analyzed with an ID Screen Flavivirus Competition ELISA (cELISA), which detects antibodies to several flaviviruses. This ELISA confirmed 96 % of WNV-, 94.7 % of TBEV- and 97 % of WNV & TBEV-BMBA-positive sera. Further testing was done with an Ingezim West Nile Compac blocking ELISA (bELISA), which mainly detects antibodies against WNV and USUV [3,4], and occasionally reacts to TBEV antibody-positive sera in mammals [5]. The bELISA confirmed most BMBA WNV-positive (47 out of 50) and WNV- & TBEV- positive sera (31 out of 33), but only a small fraction of BMBA TBEV-positive sera (37 of 286), as expected (Fig. S2; Table S4, Supplementary material 1). A subset of 12 BMBA WNV- and/or TBEV-positive sera were further analyzed with virus neutralization tests (VNT), which detected WNV- and/or TBEV- neutralizing antibodies in all tested samples (Table S6, Supplementary material 1). Among 70 BMBA WNV- & TBEV- negative sera, 36 were cELISA- and 21 bELISA-positive (Fig. S2; Table S4, Supplementary material 1). VNT analysis on 15 of these BMBA-negative sera detected WNV-neutralizing antibodies in one sample and USUV-neutralizing antibodies in four samples (Table S6, Supplementary material 1).

References

[1] C.M. Holicki, U. Ziegler, W. Gaede, K. Albrecht, J. Hänske, J. Walraph, B. Sadeghi, M.H. Groschup, M. Eiden, Tracking WNV transmission with a combined dog and wild boar surveillance system, Sci. Rep. 15 (2025) 11083. https://doi.org/10.1038/s41598-025-89561-5.

[2] R. Suluku, J. Jabaty, K. Fischer, S. Diederich, M.H. Groschup, M. Eiden, Hepatitis E Seroprevalence and Detection of Genotype 3 Strains in Domestic Pigs from Sierra Leone Collected in 2016 and 2017, Viruses 16 (2024). https://doi.org/10.3390/v16040558.

[3] E. Sotelo, F. Llorente, B. Rebollo, A. Camuñas, A. Venteo, C. Gallardo, A. Lubisi, M.J. Rodríguez, A.J. Sanz, J. Figuerola, M.Á. Jiménez-Clavero, Development and evaluation of a new epitope-blocking ELISA for universal detection of antibodies to West Nile virus, J. Virol. Methods 174 (2011) 35–41. https://doi.org/10.1016/j.jviromet.2011.03.015.

[4] U. Ziegler, F. Bergmann, D. Fischer, K. Müller, C.M. Holicki, B. Sadeghi, M. Sieg, M. Keller, R. Schwehn, M. Reuschel, L. Fischer, O. Krone, M. Rinder, K. Schütte, V. Schmidt, M. Eiden, C. Fast, A. Günther, A. Globig, F.J. Conraths, C. Staubach, F. Brandes, M. Lierz, R. Korbel, T.W. Vahlenkamp, M.H. Groschup, Spread of West Nile Virus and Usutu Virus in the German Bird Population, 2019-2020, Microorganisms 10 (2022). https://doi.org/10.3390/microorganisms10040807.

[5] M. Keller, N. Peter, C.M. Holicki, A.V. Schantz, U. Ziegler, M. Eiden, D.D. Dörge, A. Vilcinskas, M.H. Groschup, S. Klimpel, SARS-CoV-2 and West Nile Virus Prevalence Studies in Raccoons and Raccoon Dogs from Germany, Viruses 14 (2022). https://doi.org/10.3390/v14112559.
